# MCM2 promotes the stemness and sorafenib resistance of hepatocellular carcinoma cells via hippo signaling

**DOI:** 10.1038/s41420-022-01201-3

**Published:** 2022-10-15

**Authors:** Xin Zhou, Jianzhu Luo, Haixiang Xie, Zhongliu Wei, Tianman Li, Junqi Liu, Xiwen Liao, Guangzhi Zhu, Tao Peng

**Affiliations:** 1 Department of Hepatobiliary Surgery, The First Affiliated Hospital of Guangxi Medical University, Nanning, 530021 Guangxi Zhuang Autonomous Region, People’s Republic of China; 2 Guangxi Key Laboratory of Enhanced Recovery after Surgery for Gastrointestinal Cancer, 530021 Nanning, People’s Republic of China

**Keywords:** Oncogenes, HIPPO signalling, Cancer stem cells

## Abstract

Object: A large number of studies have suggested that stemness is an essential mechanism for drug resistance, metastasis and relapse in hepatocellular carcinoma (HCC). The aim of this study was to determine the impact of MCM2 on stemness and identify potential mechanisms that complement the stemness regulatory network in HCC. Methods: MCM2 expression features and prognostic significance were analyzed in multiple cohorts, including TCGA LIHC dataset, GSE14520 dataset, Guangxi cohort, and GSE76427 dataset. Stemness-related molecules and phenotypes were examined to evaluate the impact of MCM2 on stemness. The expression levels of key molecules of the hippo signaling pathway together with downstream target genes were examined to evaluate the effect of MCM2 on hippo signaling. This was further demonstrated by rescue experiments with hippo signaling pathway inhibitors (super-TDU). Sorafenib-resistant cells were constructed to assess the effect of MCM2 on drug resistance. A xenotransplantation model of nude mice was constructed to validate the role of MCM2 in vivo. Results: MCM2, which is expressed at higher levels in HCC tissue than in normal liver tissues, is a good indicator for distinguishing tumor tissues from normal liver tissues and can help differentiate HCC patients at different BCLC stages. The annotation of the differentially expressed genes in the MCM2 high and low expression groups indicated that MCM2 may be associated with the hippo signaling pathway. In addition, the expression of MCM2 in HCC tissues was correlated with the expression of YAP1/TAZ, which are key molecules of the hippo signaling pathway. It indicated that manipulation of MCM2 expression affects hippo signaling and stemness, while the inhibition of hippo signaling significantly reversed the effect of MCM2 on stemness. Disruption of MCM2 expression significantly elevated the sensitivity of sorafenib-resistant cells to sorafenib, as evidenced by the decrease in IC50 and diminished sphere-forming capacity. The in vivo assays showed that MCM2 effectively enhanced the efficacy of sorafenib. Conclusion: MCM2 is a good prognostic marker. MCM2 enhances the stemness of HCC cells by affecting the Hippo signaling pathway, while the downregulation of MCM2 inhibits resistance towards sorafenib.

## Abstract (web summary)

Long-term intervention with sorafenib induces hyper-expression of YAP thereby enhancing cell stemness and promoting the resistance. In our study, we found that in hepatocellular carcinoma tissues, MCM2 expression was highly correlated with YAP expression, and that MCM2 interference inhibited the Hippo signaling pathway by blocking YAP entry into the nucleus, thereby increasing cellular resistance to sorafenib.

## Graphical abstract

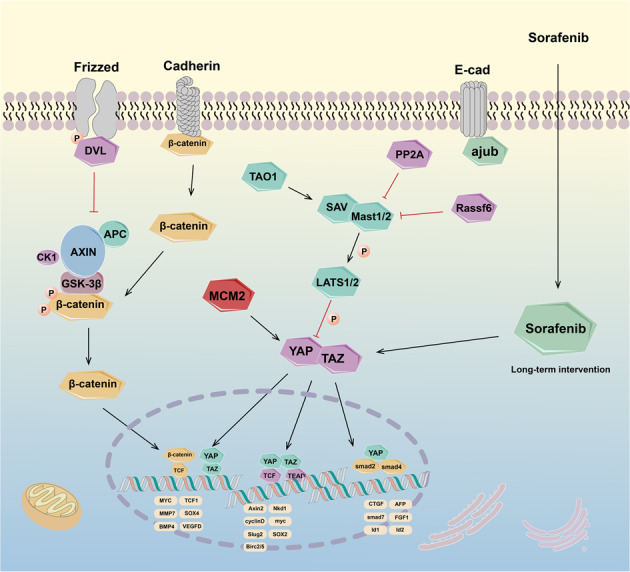

## Introduction

Commonly known for its high rates of malignancy, heterogeneity, and incidence, liver cancer is one of the most common malignancies worldwide [1–3]. Hepatocellular carcinoma (HCC) is the predominant pathological type of liver cancer, and accounts for 90–95% of all liver cancers [4]. In global terms, the incidence of liver cancer ranks 6th among all malignancies, while its mortality rate ranks even higher, taking the 4th place [5]. The imbalance between its incidence and mortality reflects its poor prognosis [6, 7]. The prevalence of HCC is particularly severe in China, which has one-fifth of the world’s population. China has an annual incidence of about 400,000 HCC cases, which is almost half of the total number of HCC cases reported worldwide [8, 9]. Hepatitis B virus (HBV), hepatitis C virus (HCV), excessive alcohol consumption, non-alcoholic steatohepatitis, long-term intake of aflatoxin-contaminated food, and various other causes of cirrhosis have been clearly identified as risk factors for HCC [10]. The lack of effective measures leads to a very poor prognosis, with 60–70% of HCC patients being diagnosed at an advanced stage and a median survival duration of only 9 months and a 5-year survival rate of about 20% [11–13]. Early detection, early diagnosis, and early treatment are key to improve the prognosis of liver cancer. In recent years, notable breakthroughs have been made for the treatment of HCC [13, 14]. Although these emerging regimens have highlighted the current situation of the treatment of patients with advanced HCC, approximately half of the patients are still classified as non-responders after treatment or disease progression continues after initial response due to drug resistance, as there are no validated biological markers that can be used to predict or monitor the efficacy of dosing. Therefore, research into methods of treatment for HCC still has a long way to go.

The concept of tumor stem cells was first introduced by Prof. Mackillop in 1983 and refers to a specific group of cells in tumors that have stem cell properties and high tumorigenicity [15]. First evidence of the presence of CSCs found that the CD34+/CD138− subpopulation in AML cells was capable of triggering tumorigenesis in NOD/SCID mice [16]. The presence of CSCs has been demonstrated in many solid tumors, including HCC. Tumor stem cells are able to expand to maintain their own cellular pool and maintain the ability of the tumor to renew itself indefinitely [17]. Liver cancer stem cells (LCSCs) are closely associated with the development, recurrence, metastasis, and drug resistance of hepatocellular carcinoma [17]. CD133, CD90, CD44, CD24, CD13, and EpCAM are considered as surface markers of CSCs in hepatocellular carcinoma [17–19]. The proportion of CSCs is higher in sorafenib-acquired resistant HCC than in sorafenib-sensitive HCC [20–22]. Nanog^+^ cells have been observed to be more resistant to sorafenib and cisplatin than Nanog− cells. The relationship between CSCs and EMT is very close, with cells with an EMT phenotype often having CSC features and in turn, CSC cells commonly have mesenchymal-like features [23, 24]. EMT activation in Sorafenib-resistant cells is often accompanied by the subpopulation enrichment of CSCs.

Research on hepatocellular carcinoma stem cells may help provide potential therapeutic directions for improving patient prognosis and improving drug tolerance. We observed significant differences in microchromosome maintenance protein 2 (MCM2) expression in HCC versus paraneoplastic tissue in previous studies, as well as its prognostic value [25]. Moreover, our study suggested that MCM2 is very closely associated with the cell cycle, DNA damage repair, and drug resistance [25]. MCM2, a member of the MCM family, is an essential regulator of eukaryotic DNA replication and drives the formation of the pre-replication complex, which is a critical first step in the G1 phase [26]. It has been demonstrated by several researchers that MCM2 was superior in terms of cell proliferation studies, compared with ki-67, in studies conducted on colon cancer, lung cancer, gallbladder adenocarcinoma, cervical cancer, and other precancerous lesions of the epithelial tissue [27–32]. MCM2 is widely known as a downstream component of the P53 pathway, and several studies have shown that P53 is involved in the regulation of stemness, indicating that MCM2 may also play a role in the maintenance of stemness characteristics and self-renewal of tumor stem cells [33–35]. Yang et al. found that MCM2 was one of the most significantly expressed proteins between HCC and paraneoplastic tissue, and that the NIR-II probe targeting MCM2 and CH1055-MCM2 could effectively identify both neoplastic and recurrent HCC in mice [36]. Based on the previous findings by our team and other researchers, this study attempted to further explore the role of MCM2 for the stemness and self-renewal ability of hepatocellular carcinoma cells and the regulatory mechanisms involved.

## Materials and methods

### Patient samples

The tissue specimens were acquired from 56 HCC patients who were operated on at the First Affiliated Hospital of Guangxi Medical University (GXMU) from August 2016 to October 2018. Tissue sections from 30 patients with HCC at the Yulin First People’s Hospital were collected and named as the Yulin cohort. In addition, tissue sections of 40 HCC patients from Liuzhou People’s Hospital formed the Liuzhou cohort. All the HCC patients have never received chemotherapy, systemic treatment, TACE (transcatheter arterial chemoembolization) or ablation before surgery resection. All patients provided written informed consent prior to being operation on. This study was approved by the Ethics Committee of the First Affiliated Hospital of Guangxi Medical University (NO.2022-KY-E-159).

### Data from public databases

Transcriptomic and clinical data of 360 patients with HCC was obtained from TCGA database through the official website of the National Cancer Institute (NCI) (https://www.cancer.gov/about-nci/organization/ccg/research/structural-genomics/tcga) [37]. The GSE14520 dataset and GSE76427 dataset were obtained from the GEO database (https://www.ncbi.nlm.nih.gov/gds/) [38].

### Cell lines and cell culture

THLE-2, MIHA, Hep3B and Huh7 cells were purchased from the National Collection of Authenticated Cell Cultures (https://www.cellbank.org.cn/). SNU-449, SNU-182, and SNU-387 were purchased from ATCC: The Global Bioresource Center (ATCC, https://www.atcc.org/). PLC/PRF/5 and MHCC-97H were provided by Prof. Lu Guo-dong of the School of Public Health, Guangxi Medical University. Hep3B was cultured in MEM medium (Gibco, Shanghai, China) supplemented with 10% FBS (Gibco, Shanghai, China). THLE-2 was cultured in BEGM medium (Lonza, CC3170, Hong Kong). SNU-449, SNU-182, and SNU-387 were cultured in RMPI-1640 medium (Gibco, Shanghai, China) supplemented with 10% FBS (Gibco, Shanghai, China). MIHA, LM3, Huh7, PLC5, and MHCC97-H were cultured in DMEM medium (Gibco, Shanghai, China) supplemented with 10% FBS (Gibco, Shanghai, China).

### Cell transfection and Lentivirus infection

The small/short interfering RNA (siRNA) designed for MCM2 interference was synthesized by Hanbio (https://www.hanbio.net/cn) and the sequences used are shown in Table S1. On the contrary, the vector carrying the wild-type full-length human MCM2 cDNA was transfected into HCC cells for MCM2 upregulation. The siRNAs and vectors were both transfected into the cells using a Lipofectamine 3000 system (Invitrogen, USA) according to the manufacturer’s instructions.

To achieve stable and durable MCM2 expression interference in the HCC cells, the sh-MCM2 lentivirus was constructed and packaged. The functional sequences of sh-MCM2 lentivirus refer to hs-MCM2-si1 and hs-MCM2-si3 in Table S1. The cells were infected with the lentivirus in the culture medium along with 10% serum according to the manufacturer’s instructions (Hanbio, Shanghai, China). After 24 h of infection, the HCC cells were cultured in a medium containing 5 μg/ml puromycin for 7 days.

### Spheroid formation assay

1000 cells were implanted into the 96-well ultralow attachment plate (Corning 3474, MA, USA) and cultured in advanced DMEM/F12 medium (Life Technologies, USA) supplemented with 100 IU/ml penicillin (Technologies, USA), 100 μg/ml streptomycin (Technologies, USA), 30 ng/ml human recombinant epidermal growth factor (Peprotein, Hong Kong), 10 ng/ml human FGF-basic recombinant protein (Life Technologies, USA), 10 ng/ml recombinant human HGF (Peprotein, Hongkong), 1% nonessential amino acids (Sigma-Aldrich, Germany), 1% GlutaMax, 2% B27 supplement (Life Technologies, USA), and 1% methylcellulose (Sigma-Aldrich, Germany). The cells were cultured in the cultivation system described above for 5 days to shape the primary spheroid. The number of primary spheres in each well were counted in 5 random fields under a microscope. Subsequently, the primary spheres were dissociated, resuspended and implanted into the new 96-well ultra-low attachment plate to generate the secondary spheroids. The number of secondary spheres were also reckoned ditto. For the spheroid formation assay, the data are displayed as the mean ± SD of the experiment performed in triplicate.

### Extreme limiting dilution analysis (ELDA)

ELDA is particularly useful for analyzing limited dilution data generated through stem cell research. It was designed to test whether different cultures have the same proportion of active cells. The cells were seeded into ultra-low adsorption plates at a density of 1, 5, 10, 15, 20, and 25 cells per well. After 7 days, each well was examined to determine tumor-sphere formation. The data of limiting dilution assays were analyzed using software available at http://bioinf.wehi.edu.au/software/elda.

### Polymerase chain reaction (PCR)

A PrimeScript™ RT reagent Kit with gDNA Eraser (Takara, Dalian, China) was used for genomic DNA erasure and reverse transcription. A TB Green® Premix Ex Taq™ (Takara, Dalian, China) was applied for the PCR assay following the manufacturer’s instructions. The target sequences of the primer are provided in Table S2. All the PCR experiments were performed on a QuantStudio 6 Flex system (Themo fisher scientific, USA).

### Immunohistochemistry (IHC)

IHC was performed using a PV-9000 system following the manufacturer’s instructions and general process followed was as previously described [39]. All primary antibodies were diluted according to the manufacturer’s instructions and incubated overnight at 4 °C. The primary antibodies used in this study were as follows: MCM2 (1:500, Proteintch, China), YAP (1:200, Proteintch, China), and MYC (1:200, Proteintch, China).

### Immunofluorescence (IF)

The cells were fixed using 4% paraformaldehyde for 20 min and Triton was used for 20 min to permeabilize the cell membranes. After fixation and permeabilization, the cells were incubated with primary antibodies for 1 h at room temperature, followed by incubation with fluorescence-labeled secondary antibodies, at concentrations recommended by the manufacturer. The primary antibodies used in this study were YAP and MCM2. DAPI dyes were used for the development of the cell nuclei. All experiments were performed in triplicate.

### Western blotting analysis

The protocol used to conduct the western blotting assay are given in our previous study [39]. The concentrations of the primary antibodies were as follows: MCM2 (1:5000, 66204-1-Ig, Proteintech, China), β-actin (1:20000, 81115-1-RR, Proteintech, China), CD133 (1:3000, 66666-1-Ig, Proteintech, China), Bmi1 (1:1000, 66161-1-Ig, Proteintech, China), Epcam (1:1000, 66316-1-Ig, Proteintech, China), ALDH1A1 (1:500, 15910-1-AP, Proteintech, China), AKT1 (1:5000, 60203-2-Ig, Proteintech, China), c-myc (1:5000, 67447-1-Ig, Proteintech, China), LaminA/C(1:5000, 10298-1-AP, Proteintech, China), and SOX9 (1:2000, 67439-1-Ig, Proteintech, China). After incubation with the primary antibodies at 4 °C overnight, the PVDF membrane was incubated with the corresponding goat anti-mouse or goat anti-rabbit antibody. All experiments were performed in triplicate.

### CCK-8 assay

First, 1000 cells were seeded into 96-well plates, with 6 replicates set up in each group. Five identical plates were inoculated on the first day and placed in the cell incubator. One plate was taken out each day, CCK-8 reagent was added to each well, and absorbance at 488 nm was measured after two hours of incubation. After an interval of 24 hours, the next plate was removed and the same procedure described above was performed to detect the absorbance. The growth curves of each group of cells were plotted on GraphPad prism 8 software based on the results of 5 days of experiments.

### Colony formation assay

To assess the impact of MCM2 on colony formation and Lenvatinib resistance evaluations, colony formation assays were performed as described in our previous study [39]. The indicated cells were seeded at a density of 4000 cells per well into 6-well plates. All experiments were performed in triplicate. For the colony formation assay, the data are displayed as the mean ± SD of three wells.

### Flow cytometry

After digestion and resuspension, the cells were stained using the Cell cycle detection Kit (Keygen, Jiangsu, China) and were subsequently placed on a Flow cytometer to detect the dyeing strength and proportion. The exact process followed has been described previously [39]. All experiments were performed in triplicate. The data are displayed as the mean ± SD of three groups.

### Gene set enrichment analysis (GSEA)

GSEA performs the functional annotation of genes based on prior knowledge using a hypergeometric enrichment algorithm. The gene data source is the differential genes obtained by the experimental group vs the control group, and the differential genes were determined based on threshold P and FDR values. The detailed implementation method is provided in our previous research study [40].

### Tumor xenograft models

Male nude mice of 4 weeks of age were used for the animal experiments in this study. After digested with EDTA-trypsin (Solarbio, Beijing), the cells in the sh-MCM2 group and sh-NC group of MHCC-97H were resuspended in PBS (Solarbio, Beijing) and were made into a homogeneous single cell suspension. The cells were diluted to a concentration of 1 × 10^8^ per milliliter, and 200 µl of the cell suspension was subcutaneously injected into the axilla of each nude mouse. Based on the injected cells, the 8 nude mice in the sh-MCM2 group and the 8 nude mice sh-NC group were respectively divided into two groups. The sample size of nude mice was estimated according to our previous study [39]. Then, 2 weeks later, the tumor xenografts were subcutaneously formed in the nude mice. Subsequently, in each group, half of the nude mice were randomly selected and intraperitoneally injected with sorafenib (5 mg/kg), while the other half were injected with an equal volume of the placebo. The nude mice were divided into four groups based on MCM2 manipulation and sorafenib/placebo intraperitoneal injection. Tumor growth in nude mice was kept under observation and growth was recorded. The volume of each tumor was calculated using the formula: V = length × width^2^× π/6.

The MHCC-97H cells of the sh-MCM2 group and sh-NC group were collected, and the cell concentration was adjusted to 1 × 10^7^/mL using PBS. Then, 200 μL cell suspension was injected into the tail vein of the nude mice. Two weeks later, 12 nude mice in sh-MCM2 group and 12 nude mice sh-NC group were randomly divided into two groups and administered with an intraperitoneal injection of sorafenib (5 mg/kg) or the placebo. The sample size of nude mice was estimated according to previous study [41]. Four weeks after MHCC-97H cell inoculation, the nude mice were sacrificed, and the lung tissues were isolated. The lung tissues were fixed using formalin and were sliced. HE staining was performed to observe the number of metastases and compare differences in the number and diameter of metastases between the indicated groups.

### Statistical analysis

The program G*Power 3.1.9 was used to estimate the sample size and statistical power. An a priori power analysis was performed to estimate sample size with power of 0.95 and alpha value of 0.05. Under the background of no significance in F-test and in accordance with normal distribution, Student’s t test was applied to analyze differences in MCM2 expression between HCC and para-carcinoma tissues. Under the background of no significance in F-test, ANOVA was used to inspect differences in MCM2 expression among HCC patients at different stages. The Kaplan-Meier method was used to conduct the survival analysis. The data on the limiting dilution assays were analyzed using software available at http://bioinf.wehi.edu.au/software/elda. Each test was performed in triplicate. A P value of <0.05 was considered to indicate statistical significance.

## Results

### MCM2 is upregulated in HCC tissues is associated with a poor prognosis

**Fig. 1:**
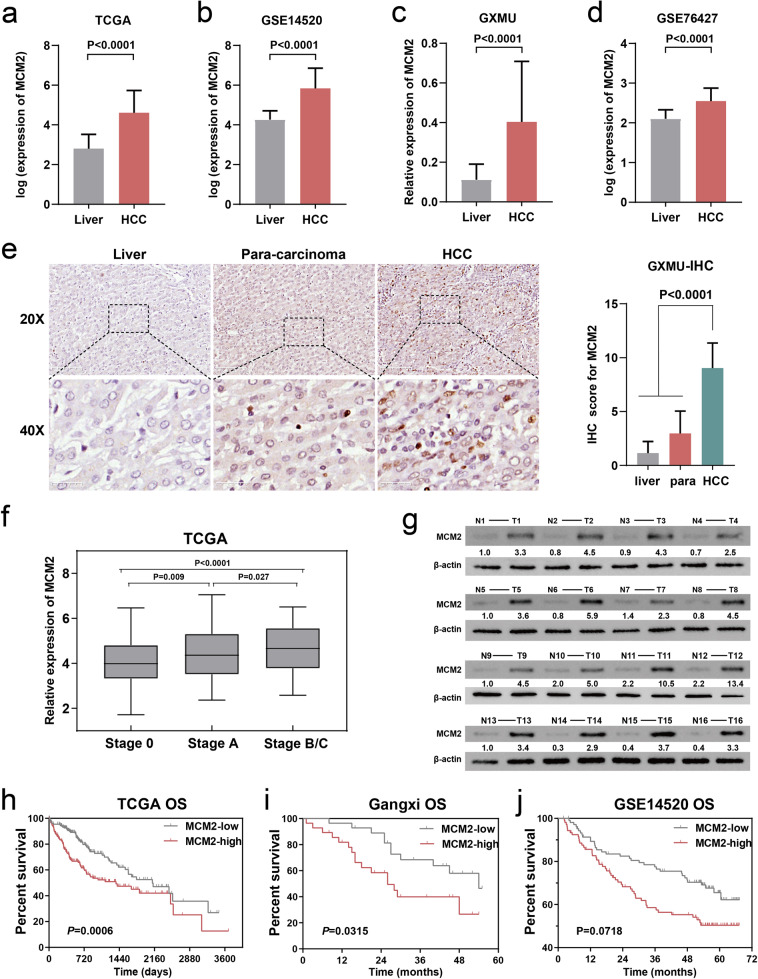
MCM2 is high expressed in HCC tissues and is associated with poor prognosis. **a–d** Expression of MCM2 at the transcription level between HCC and normal liver tissues in TCGA LIHC dataset, GSE14520 dataset, GXMU cohort and GSE76427, and differences of MCM2 expression were statistically significant in each cohort; **e** Expression of MCM2 in HCC, para-carcinoma and normal liver tissues in GXMU cohort determined by immunohistochemistry assay, and the intensity of MCM2 expression was significantly higher in HCC than in para-carcinoma and liver tissue; **f** The expression of MCM2 in HCC patients with different BCLC stages in TCGA LIHC dataset, and patients in advanced BCLC stage were with higher expression of MCM2; **g** The immunoblotting for MCM2 expression of HCC tissues (T) and normal liver tissues (N) in GXMU cohort, determined by Western Blot assay; **h–j** Using the median MCM2 as the cut-off value, the prognosis of patients with high and low expression HCC differed significantly, with a poor prognosis for those with high MCM2.

Primarily, the differences in the expression of MCM2 and its trend in cancer tissues and paraneoplastic tissues were explored, along with the relationship between MCM2 and clinicopathological and prognostic features to determine a preliminary understanding of the potential oncogenic role of MCM2 in HCC. The clinicopathological information on the 56 HCC patients in the GXMU are presented in Table 1, and these patients were classified into either a high or low expression groups based on the median MCM2 expression level. At the transcriptome level, the results of expression analysis of multiple cohorts, including TCGA LIHC dataset, GSE14520 dataset, GSE76427 dataset, and GXMU cohort, all consistently suggested that the expression of MCM2 in HCC tissues is significantly higher than that of liver tissues (Fig. 1a–d). To compare the expression of MCM2 at the protein level, immunohistochemistry was performed using the HCC and paraneoplastic tissue sections of patients from the First Affiliated Hospital of Guangxi Medical University (Fig. 1e), Yulin First People’s Hospital (Fig. S1a), and Liuzhou People’s Hospital (Fig. S1b). The IHC results of these cohorts all suggested that MCM2 expression in cancer was significantly higher than that of the paraneoplastic tissues, and that MCM2 expression in paraneoplastic tissue was also higher than that of normal liver tissues. In addition, MCM2 was found to be mainly distributed in the nucleus of the HCC cells, which suggested that the nucleus might be the location at which the MCM2 protein plays its role. Additionally, there are statistically significant differences in MCM2 expression between patients at different clinical stages, and MCM2 expression was found to have increased along with the progression of HCC (Fig. 1f). Using western blotting assays, we quantitatively analyzed MCM2 expression in HCC and para-carcinoma tissues of 16 patients at the First Affiliated Hospital of Guangxi Medical University. The expression of MCM2 was very low in normal tissue but was more abundant in HCC tissues (Fig. 1g). Considering the significant difference in expression, we attempted to analyze the diagnostic performance of MCM2 for HCC. We performed a ROC analysis using multiple cohorts, including TCGA LIHC dataset, GSE14520 dataset, GSE76427 dataset, and data of the First Affiliated Hospital of Guangxi Medical University. The results of the ROC analysis of these cohorts showed that the AUC of the ROC in each cohort was greater than 0.800, while the AUC of the ROC of TCGA, GSE14520, and GSE76427 were even greater than or equal to 0.900 (Fig. S1c). The difference in MCM2 expression between the HCC and liver tissues can be used to effectively distinguish cancer tissue from paraneoplastic cancer tissue. Finally, we explored the association between MCM2 and the prognosis of HCC using the K-M method in terms of survival data obtained from TCGA LIHC dataset (Fig. 1h), the dataset from the First Affiliated Hospital of Guangxi Medical University (Fig. 1i), and the GSE14520 dataset (Fig. 1j). Analysis of all three cohorts suggested that MCM2 was strongly associated with the prognosis of HCC, and that patients with a high level of expression showed a worse overall prognosis.

**Table 1:** Basic information of 56 HCC patients from the first affiliated hospital of GXMU.

Variables	Number of cases	MCM2 expression		X^2^	P value
		low	high		
Age(years)					
<60	41	20	21		
>=60	15	8	7	0.091	0.763
Gender					
male	53	28	25		
female	3	0	3	NA	0.236
BMI					
<25	42	21	21		
>=25	14	7	7	0	1
Alcohol					
no	38	20	18		
yes	18	8	10	0.327	0.567
Cirrhosis					
no	3	1	2		
yes	53	27	26	NA	1
Child pugh					
A	54	28	26		
B	2	0	2	NA	0.491
BCLC stage					
A	45	26	19		
B/C	11	2	9	5.543	0.019
AFP					
<200 ng/mL	31	17	14		
>=200 ng/mL	24	11	13	0.493	0.508
missing	1				
Residue radical					
R0	47	25	22		
R1	9	3	6	NA	0.469

*HCC* Hepatocellular Carcinoma, *GXMU* Guangxi medical university, *BMI* Body Mass Index, *BCLC* barcelona clinic liver cancer.

### Impact of the regulation of MCM2 on the stemness of HCC cells

**Fig. 2:**
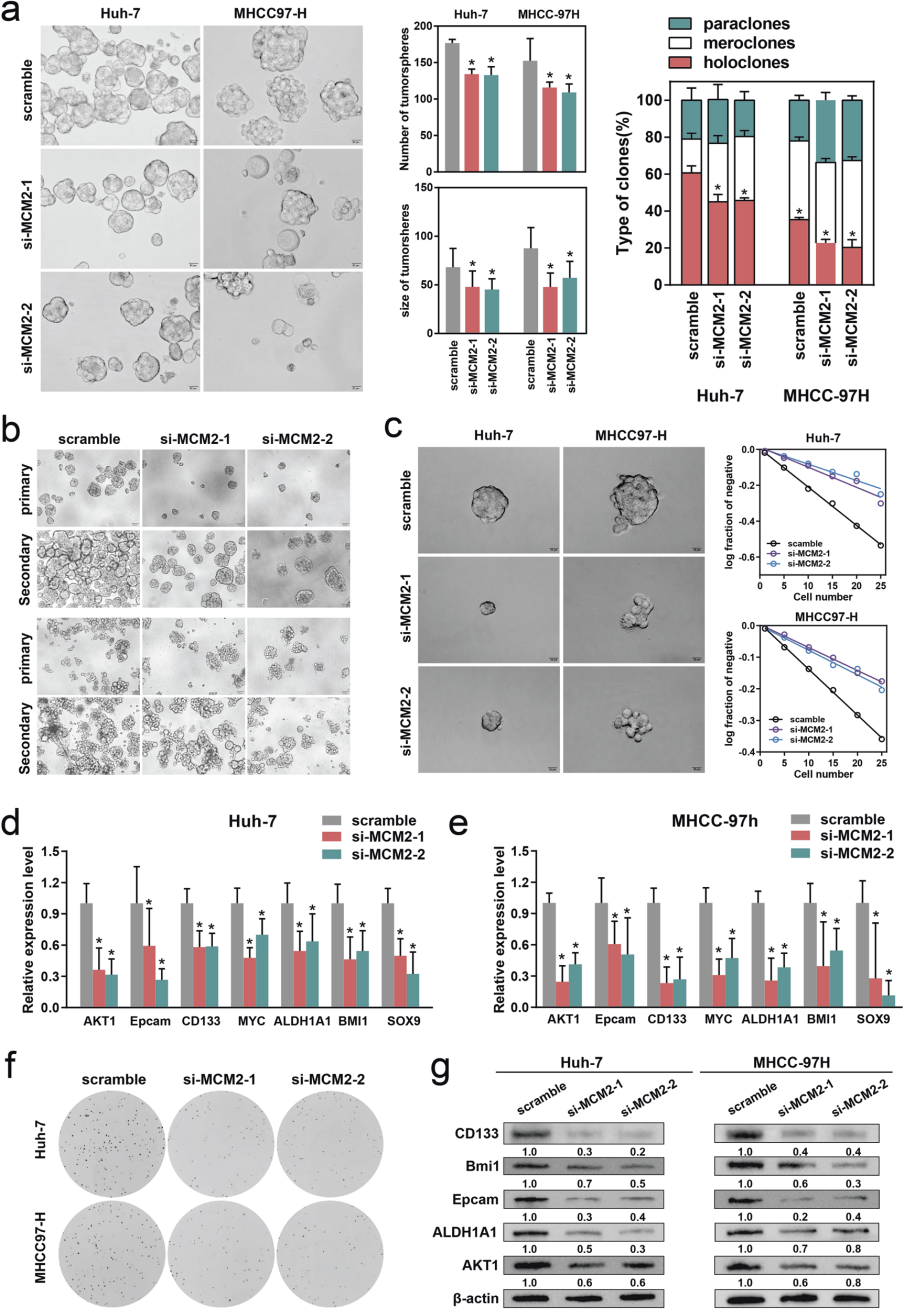
Downregulation of MCM2 weakened the stemness of HCC cells. **a** Representative images of tumor-spheres in the si-MCM2 and scramble groups of the indicated cells, corresponding histogram and corresponding sphere morphology percentage chart; **b** Representative images of primary and secondary spheres formation in the si-MCM2 and scramble groups of the indicated cells, with corresponding histogram displayed in Fig S2d; **c** Representative images of tumor-spheres formation in the limiting dilution assays of the indicated cells and the log-dose slope of the si-MCM2 and scramble groups. **d, e** Expression of HCC stem biomarkers, including AKT1, Epcam, CD133, MYC, ALDH1A1, BMI1 and SOX9, in the si-MCM2 and scramble groups of the indicated cells. **f** Representative images of colony in the si-MCM2 and scramble groups of the indicated cells, with corresponding histogram displayed in Fig. S2e. **g** The immunoblotting for Bmi1, AKT1, CD133, ALDH1A1 and Epcam expression in the si-MCM2 and scramble groups of the indicated cells, determined by Western Blot assay; Data are shown as the mean ± SD from independent experiments. *P < 0.05.

The expression levels of MCM2 in several hepatoma cell lines and normal hepatocyte lines were examined using qPCR. Apart from THLE-2 and MIHA in the normal hepatocyte cell lines, the MCM2 expression levels were the highest in MHCC-97H and HUH-7, while the lowest was recorded in SNU-449 and Hep3B (Fig. S2a). Complementary siRNAs were used to knockdown the expression of MCM2 in MHCC- 97H and HUH-7. Plasmids carrying the wild-type MCM2 sequences were used to achieve upregulation of MCM2 expression levels in the SNU-449 and Hep3B cells (Fig. S2b, c). The amplification effect and knockdown effect were evaluated by performing qPCR and western blotting (Fig. S2b, c). In the MHCC-97H and HUH-7 cells, after 5 days of spheroid formation, the volume and number of spheroids in the si-MCM2 group decrease significantly compared with the scramble group. In addition, the proportion of spheroids with a clonal morphology in the si-MCM2 group was altered, as shown by the decrease in the proportion of holoclones, as well as the increase in the proportion of paraclones and meroclones (Fig. 2a). Primary spheroid-forming cells were replanted in ultra-low adsorption culture plates to achieve a second spheroid formation. The number and diameter of the spheroids in the si-MCM2 group were significantly smaller than that of the scramble group during both primary and secondary spheroid formation (Fig. 2b), and the corresponding histograms are shown in Fig. S2d. Limitation dilution assays were conducted to evaluate the self-renewal ability and stemness of the cells. In MHCC-97H and HUH-7 cells, suppression of MCM2 significantly restrained the formation of tumor spheroids, and thus the probability of negative responses increased in the knockdown group. Therefore, the log (fraction of negative) value increased and the slope of the curve increased in the si-MCM2 group under the same number of implants (Fig. 2c). The colony formation assay assessed the colony-forming ability of the cells on a flat surface, which is also a reflection of cell stemness. After being grown with the same number of implants, under the same culture conditions, significantly fewer colonies were formed in the si-MCM2 group, compared with the scramble group (Fig. 2f), with the corresponding histogram displayed in Fig. S2e. After confirming the effect of MCM2 suppression on the self-renewal ability and stemness of the cells, changes in the expression levels of the stemness-related molecules were further examined. The qPCR assays confirmed that the suppression of MCM2 caused a decrease in the expression of AKT1, Epcam, CD133, MYC, ALDH1A1, BMI1, and SOX9 in the MHCC-97H and Huh7 cells (Fig. 2d, e). The effects of MCM2 on stemness was also assessed by performing western blotting assays, and a decrease in stemness was observed in the si-MCM2 group (Fig. 2g).

**Fig. 3:**
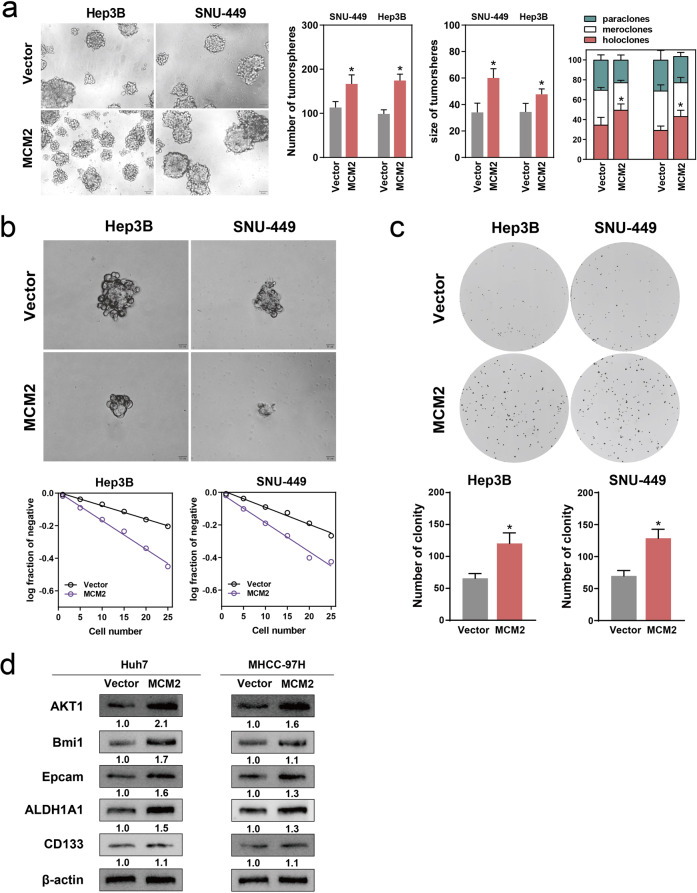
The up-regulation of MCM2 enhanced the stemness of HCC cells. **a** Representative images of tumor-spheres in the MCM2 over-expression and control groups of the indicated cells, corresponding histogram and corresponding sphere morphology percentage chart. **b** Representative images of tumor-spheres formation in the limiting dilution assays of the indicated cells and the log-dose slope of the MCM2 over-expression and control groups. **c** Representative images of colony in the MCM2 over-expression and control groups of the indicated cells and corresponding histogram; **d** The immunoblotting for Bmi1, AKT1, CD133, ALDH1A1 and Epcam expression in the Vector and MCM2 groups of the indicated cells, determined by Western Blot assay; Data are shown as the mean ± SD from independent experiments. *P < 0.05.

We proceeded to examine the effect of MCM2 on the cell stemness phenotype using plasmid transfection. In contrast to the effect produced by the suppression of MCM2 expression, we observed the enhanced expression of MCM2 significantly elevated the number and diameter of sphere-forming Hep3B and SNU-449 cells (Fig. 3a). The same number of seeded plants produced a significantly higher percentage of clones in the Hep3B and SNU-449 cells transfected with the MCM2 plasmid, compared with the respective vector groups (Fig. 3b). Similarly, transfection with the MCM2-plasmid resulted in the acquisition of the colony-formation potential of the Hep3B and SNU-449 cells (Fig. 3c). In addition to the effect on spherogenesis, aberrant MCM2 expression was shown to promote the expression levels of stemness-related molecular markers (Fig. 3d).

### MCM2 is implicated in the hippo signaling pathway

**Fig. 4:**
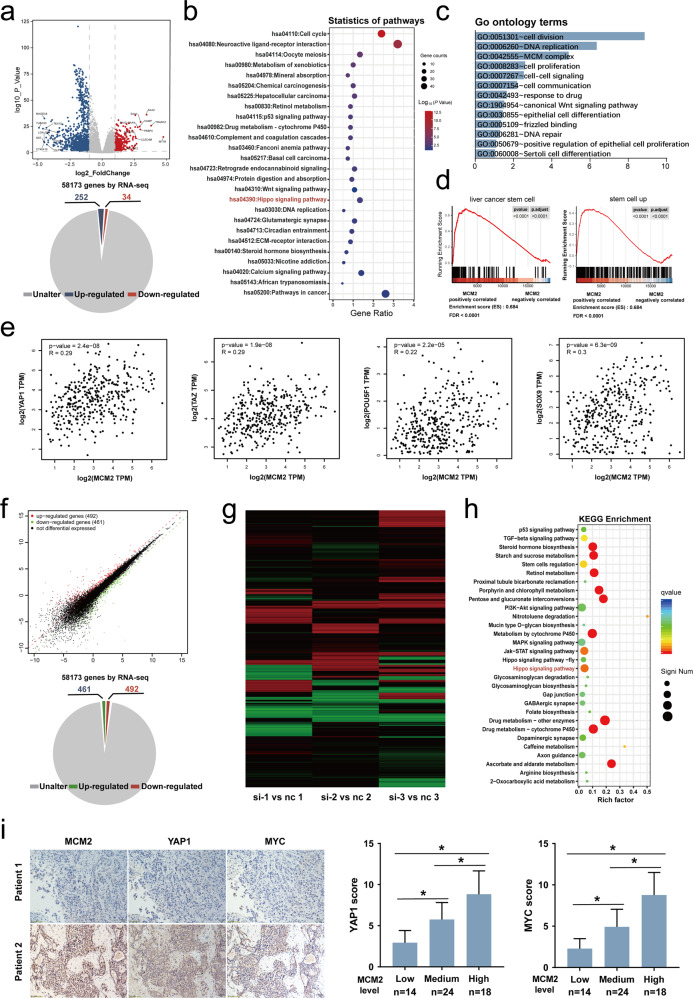
Transcriptome analysis of HCC tissues and HCC cells both suggested that MCM2 was implicated with Hippo signaling. **a** Using the median MCM2 expression as a cut-off value, patients in the TCGA LIHC dataset were divided into two groups and the genes that differed between the two groups are presented as a heat maps; **b**, The bubble diagram showing the results of the KEGG pathway annotation for the differential expression genes mentioned hereinbefore; **c**, The bar chart showing the results of the GO terms annotation for the indicated differential expression genes; **d**, Results of GSEA based on high MCM2 expression and low MCM2 expression groups from TCGA LIHC dataset; **e** Scatter plots showing the correlation of MCM2 expression with YAP1, TAZ, Oct4 and SOX9 based on TCGA LIHC dataset. **f** Scatter plot of the differently expressed genes between si-MCM2 group and scramble group in MHCC-97H cells. **g** Heatmap of differentially expressed genes in the three paired groups for three repeated assays for MCM2 interference. **h,** The bubble diagram showing the results of the KEGG pathway annotation of the differential expression genes in indicated cells; **i**, Representative images of immunohistochemistry for mcm2, YAP1 and c-myc and corresponding histograms. Data are shown as the mean ± SD from independent experiments. *P < 0.05.

TCGA LIHC of the 360 HCC patients were divided into whether a high or low expression groups based on the median expression of MCM2. Subsequently, the whole transcriptome matrices of patients in the two groups were compared and a number of differentially expressed genes were identified. Genes with a *P* < 0.05 and |log(P value)| > 1 were defined as differentially expressed genes. As shown in the pie plot and the volcano plot, a total of 286 differentially expressed genes were identified between the two groups, of which 34 were upregulated and 252 were downregulated (Fig. 4a). The KEGG pathway enrichment analysis of the differential genes indicated that MCM2 is involved in the p53 signaling pathway, cell cycle, and hippo signaling pathway (Fig. 4b); Moreover, hepatocellular carcinoma (hsa05225) appeared in the results of the enrichment analysis, indicating that MCM2 plays a key role in the development and progression of HCC (Fig. 4b). Using the GO (Gene ontology) database for comparison, the enrichment analysis of the differential genes found that MCM2 is associated with biological processes, such as cell division, DNA replication, cell expansion, and MCM complex formation (Fig. 4c). The GSEA analysis of TCGA database suggested that MCM2 is associated with stem cells and liver cancer stem cells (Fig. 4d). GEPIA (http://gepia.cancer-pku.cn/) is a website used to perform gene expression profiling interactive analysis based on TCGA database. GEPIA was used to explore the expression between MCM2 and major molecules of the hippo signaling pathway. The analysis based on GEPIA indicated that there is a high level of correlation between the expression of MCM2 and several molecules of the hippo pathway, including POU5F1, SOX9, TAZ, and YAP1 (Fig. 4e).

Following MCM2 knockdown, Huh-7 cells were used for transcriptomic sequencing, and 492 upregulated and 461 downregulated genes were identified in the si-MCM2 group, compared with the control (Fig. 4f). The distribution of the differential genes in the three replicate samples were subjected to transcriptome sequencing and the results are presented as a heat map (Fig. 4g). Enrichment analysis of the differential genes showed that MCM2 is associated with the Hippo signaling pathway, which was consistent with the enrichment analysis based on TCGA (Fig. 4h).

IHC was performed on HCC tissues to detect the expression of MCM2, MYC and YAP1 (Fig. 4i). Based on the IHC score, MCM2 expression was classified as low, medium or high. The expression levels of YAP1 and MYC were respectively significantly different among low, medium, or high groups (Fig. 4i).

### Hippo signaling repression reversed enhanced stemness induced by MCM2

**Fig. 5:**
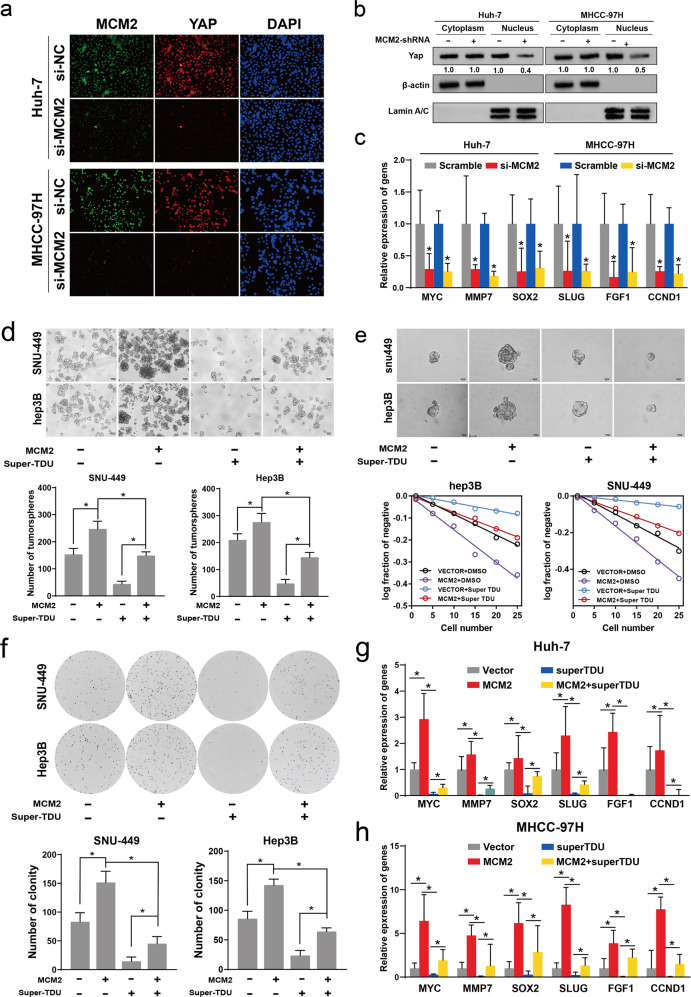
Molecular inhibitors of hippo signaling reverse the enhancement of stemness by MCM2. **a** Representative immunofluorescence images for MCM2 and YAP1 in indicated cells. green for MCM2, red for YAP1 and blue for DAPI; **b** Localization and quantifying of YAP1 by analyzing the cytoplasmic and nuclear fractions of the indicated cells, with β-actin and LaminA/C used as cytoplasmic and nuclear fraction controls, respectively; **c** The mRNA expression levels of the hippo signaling targets MYC, MMP7, SOX2, Slug2, FGF1, and CCND1 in si-MCM2 and scramble groups in the indicated cells were determined by qPCR; **d** Representative images of tumor-spheres in the MCM2 over-expression and control groups treated with hippo inhibitor (Super-TDU, 2 μM)/solvent for 48 h and corresponding histogram; **e** Representative images of tumor-spheres formation in the limiting dilution assays and the log-dose slope for MCM2 over-expression and control groups treated with hippo inhibitor (Super-TDU, 2 μM)/solvent for 48 h in the indicated cells; **f** Representative images of colony in the MCM2 over-expression and control groups treated with hippo inhibitor (Super-TDU, 2 μM)/solvent for 48 h of the indicated cells and corresponding histogram; **g, h** The mRNA expression levels of MYC, MMP7, SOX2, Slug2, FGF1, and CCND1 in MCM2 over-expression and control groups treated hippo inhibitor (Super-TDU, 2 μM) or solvent for 48 h in the indicated cells were determined by qPCR; Data are shown as the mean ± SD from independent experiments. *P < 0.05.

The results of the immunofluorescence assays conducted on MHCC-97H cells and Huh-7 cells found that the expression YAP1 decreased following MCM2 suppression (Fig. 5a). Total cytoplasmic and cytosolic proteins were extracted from the MCM2 knockdown group and control cells, respectively, to determine YAP expression. YAP expression in the nucleus of the si-MCM2 group was significantly lower than that of the scramble group, in both Huh-7 and MHCC-97H cells, whereas the difference in the expression of YAP between the si-MCM2 and scramble groups in the cytoplasm was not significant, suggesting that MCM2 knockdown inhibited the entry of YAP into the nucleus (Fig. 5b). The expression of target genes of the Hippo signal pathway, such as MYC, MMP7, SOX2, SLUG, FGF1, and CCND1, decreased significantly in both Huh-7 and MHCC-97H cells following MCM2 downregulation (Fig. 5c).

The above experiments suggest that the suppression of MCM2 can regulate Hippo signaling by inhibiting the entry of YAP into the nucleus, which in turn inhibits the transcription of target genes downstream of the Hippo signaling pathway. To further verify the reliability of this deduction, a rescue assay that used Super-TDU, a specific inhibitor of YAP, was designed. Hep3B and MHCC-97H cells were divided into four groups based on whether they were transfected with the MCM2 plasmid and were with or without the intervention of Super-TDU. Then, spheroid formation assays, restriction dilution assays, and plate cloning assays were performed. The results of these assays suggested that the addition of Super-TDU reversed the effect of MCM2 on stemness and the self-renewal ability of the hepatocellular carcinoma cells (Fig. 5d–f). The expression of the target genes of the Hippo signaling pathway were also reversed by Super-TDU following MCM2 overexpression in Hep3B and SNU-449 cells (Fig. 5g, h).

### MCM2 inhibition reduced sorafenib resistance in the HCC cells

**Fig. 6:**
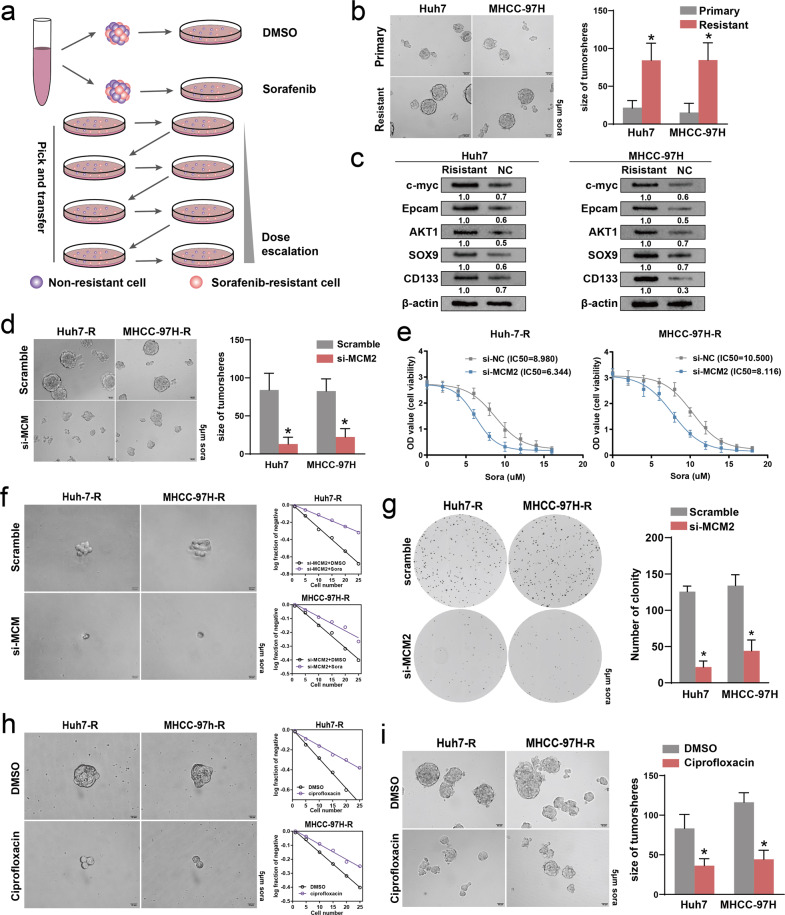
Down-regulation of MCM2 inhibit the resistance of HCC cells to Sorafenib. **a** Diagram of the construction of sorafenib-resistant cell lines; **b** Representative images of tumor-sphere for sorafenib-resistant and non-resistant groups of the indicated cells and corresponding histogram, under 5 μM sorafenib intervention. **c** Representative images of tumor-sphere for si-MCM2 and scramble groups of the indicated cells and corresponding histogram, under 5 μM sorafenib intervention. **d** Viability of si-MCM2 and scramble groups of the indicated cells under multiple concentrations of sorafenib intervention. **e** The immunoblotting for c-myc, Epcam, AKT1, SOX9 and CD133 expression in the si-MCM2 and scramble groups of the indicated sorafenib-resistant cells, determined by Western Blot assay; **f** Representative images of tumor-spheres formation in the limiting dilution assays of the indicated sorafenib-resistant cells treated with sorafenib (5 μM) and the log-dose slope of the si-MCM2 and scramble groups. **g** Representative images of colony in the si-MCM2 and scramble groups of indicated sorafenib-resistant cells treated with sorafenib (5 μM) and corresponding histogram. **h** Representative images of tumor-spheres formation in the limiting dilution assays of the indicated sorafenib-resistant cells treated with sorafenib (5 μM) and the log-dose slope of the ciprofloxacin (MCM2 inhibitor) and solvent groups. **i** Representative images of tumor-spheres in the ciprofloxacin (MCM2 inhibitor) and solvent groups of indicated sorafenib-resistant cells treated with sorafenib (5 μM) and corresponding histogram. Data are shown as the mean ± SD from independent experiments. *P < 0.05. sora, sorafenib.

Starting from a low concentration of 0.5 uM, the dose of sorafenib was gradually increased to 5uM over a 3-month period, allowing Huh7 cells and MHCC-97H cells to gradually acquire sorafenib resistance (Fig. 6a). MHCC-97H and Huh-7 cells showed a significant increase in the expression abundance of the stemness molecules after acquiring drug resistance (Fig. 6e). The stemness of the MHCC-97H and Huh-7 cells were also elevated along with the acquisition of drug resistance (Fig. 6b).

In the sorafenib-resistant HuH-7 and MHCC-97H cell lines, suppression of MCM2 expression significantly inhibited spheroid growth and cell viability (Fig. 6c, d). The results of the limiting dilution assays performed on the sorafenib-resistant MHCC-97H/Huh-7 cells showed that there was a significant reduction in the self-renewal ability and stemness following MCM2 interference, as evidenced by a reduction in the positive rate of sphere formation in the si-MCM2 groups (Fig. 6f). In addition to the 3D culture, the downregulation of MCM2 also caused changes in colony formation and clonal cluster growth in the planar culture system (Fig. 6g).

In addition to genetic inhibition, we also examined the effect of the inhibition of the pharmacology of MCM2 on the effect of sorafenib. Similar to the effect of RNA interference, the pharmacological inhibition of MCM2 also significantly enhanced the therapeutic effect of sorafenib. In sorafenib-resistant MHCC-97H/Huh-7 cells, the effect of sorafenib on inhibition of stemness and self-renewal were significantly enhanced (Fig. 6h, i).

### MCM2 suppression restrained hippo signaling and enhanced the effect of sorafenib in mice

**Fig. 7:**
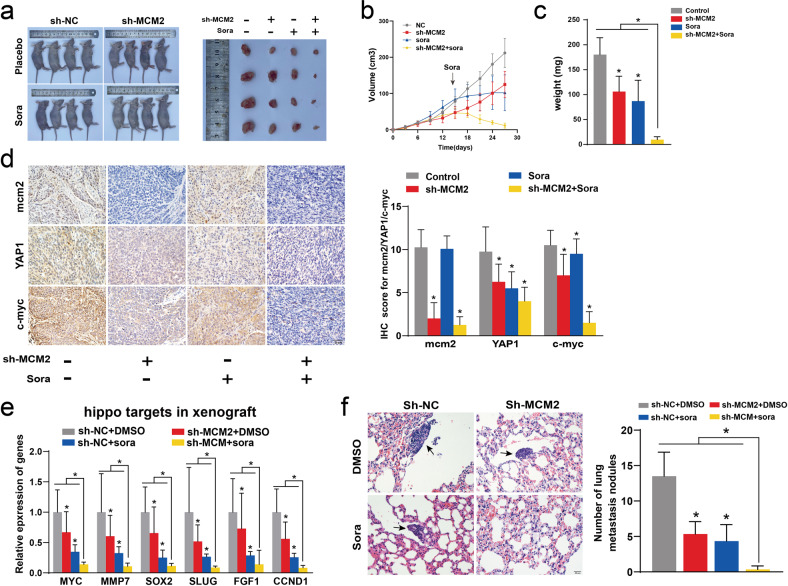
Down-regulation of MCM2 inhibited hippo signal and enhanced the efficacy of sorafenib in mice. **a** Photographs of nude mice in xenograft models and Photograph of a transplanted tumors removed from under the skin of nude mice. **b** Volume curve of the transplantation tumors during the experimental cycle, based on the diameter; **c** The weight of the transplanted tumors, when removed from the nude mice. **d** Representative images of immunohistochemistry for mcm2, YAP1 and c-myc in transplantation tumors and corresponding histograms. **e** The mRNA expression levels of MYC, MMP7, SOX2, Slug2, FGF1, and CCND1 in transplantation tumors. **f** Representative picture of pulmonary metastases after hematoxylin-eosin staining and corresponding statistical charts. Data are shown as the mean ± SD from independent experiments. *P < 0.05. sora, sorafenib.

Experiments were performed to verify whether MCM2 could enhance the efficacy of sorafenib in vivo. The effect of MCM2 on the efficacy of sorafenib was evaluated based on two aspects: the growth of the transplanted tumors and metastasis. The nude mice were divided into four groups based on the presence or absence of MCM2 interference and the presence or absence of sorafenib injection (Fig. 7a, b). The results of the xenograft subcutaneous tumorigenesis assay suggested that graft growth was limited by the interference caused by MCM2 expression, while sorafenib administration also significantly inhibited graft growth (Fig. 7c, d). Subsequently, the expression of mcm2, YAP1, and c-myc in four groups of transplanted tumors was examined by performing immunohistochemical staining. Both MCM2 interference and sorafenib administration reduced the expression of YAP1 and c-myc. In addition, targets of the hippo singling pathway were detected in the xenograft using qPCR. The reduction in the expression of target genes the hippo signaling pathway due to the effect of sorafenib combined with MCM2 downregulation was more significant, compared to the effect of each factor alone. The results of the xenograft cell lung metastasis assay showed that MCM2 downregulation and sorafenib also inhibited transplantation tumorigenesis, and that none of the nude mice in the shMCM2+sorafenib group developed lung metastasis.

## Discussion

HCC is a highly malignant and aggressive tumor, while drug resistance, recurrence, and metastasis have led to the poor prognosis of HCC patients. At the same time, HCC is highly heterogeneous with cells of different molecular subtypes found within a single lesion. Each molecular subtype exhibits completely different biological molecular properties and significant differences in multiple tumor-associated malignant biological behaviors, such as proliferation, invasion, and drug resistance.

Among HCC tumors, a smaller population of drug-resistant hepatocellular carcinoma stem cells (LCSCs) that show characteristics that are similar to somatic stem cells, which are capable of self-renewal and maintain HCC growth through asymmetric cell division. Since the introduction of the LCSCs model, researchers have recognized that the proliferation, drug resistance, and recurrence of HCC cells are mainly caused by cancer cell stemness. However, LCSCs possess multiple genomic and transcriptomic features, and these molecular patterns give LCSCs the ability to invade, self-renew, and grow independently of adhesion. Therefore, the targeting of LCSCs is the only way to achieve complete control of HCC. Since patients with advanced HCC cannot benefit from surgical species, systemic therapy is preferred. The discovery of therapies that target LCSCs have profound clinical value in improving response to systemic therapy and blocking acquired drug resistance.

For a long time, researchers understood MCM2 in terms of DNA replication and cell proliferation, and it was common perception that MCM2 is a gene downstream of the P53 pathway. MCM2 and its family members, which together form the MCM complex, play an important role in DNA replication. However, recently several researcher studies have found that in addition to DNA replication, MCM2 can also regulate transcription. In addition, an increasing number of studies have also found that MCM2 is a very sensitive indicator of proliferation, and that in some tumors application of its histopathology is even comparable to Ki-67. Tim et al. suggested that MCM2 defines the proliferative state of renal cell carcinoma and that its expression is correlated with the prognosis of renal cell carcinoma. Mar et al. found that MCM2 was more useful in distinguishing follicular carcinoma from follicular adenoma than Ki-67. Our analysis based on several cohorts of patients at the First Affiliated Hospital of Guangxi Medical University, GSE14520, and TCGA demonstrate that MCM2 is very sensitive in differentiating HCC from liver tissue. At both the transcriptional and translational levels, MCM2 expression is much higher in HCC than in normal tissues. Specimens obtained from three hospitals in Guangxi (The First Affiliated Hospital of Guangxi Medical University, Yulin First People’s Hospital, and Liuzhou People’s Hospital) showed that MCM2 expression was invariably an accurate predictor of histological type, with the proportion and intensity of MCM2 staining significantly higher in all tissue sections than next to cancer tissues. In turn, the poor prognosis of patients with high MCM2 expression demonstrates the potential pathological application of MCM2 in a clinical setting.

Our subsequent cellular assays continue to add molecular mechanistic evidence to this hypothesis, with either the pharmacological or genetic suppression of MCM2 clearly infringing on cell stemness. In contrast, exogenous supplementation of MCM2 enhanced cell stemness. These results demonstrate that MCM2 is an important link for the regulation of stemness in LCSCs. Bioinformatics and rescue assays have established that MCM2 regulates stemness of LCSCs by influencing Hippo signaling, and that the suppression of MCM2 genes significantly prevents the entry of YAP into the nucleus. Specific inhibitors of the Hippo pathway also reverse the effects of MCM2 upregulation on stemness. Thus, inhibitors that target either Hippo signaling or the MCM2 gene may be potential adjuvant therapeutic agents for clinical liver cancer.

Sorafenib currently remains the first-line option for the treatment of advanced HCC. However, due to primary drug resistance, only about 30% of patients will benefit sorafenib therapy. This population usually acquires resistance within 6 months. In most cases, sorafenib-resistant HCC cells exhibit a significant mesenchymal phenotype and stem cell characteristics. This study showed that MCM2 was significantly elevated in sorafenib-resistant HCC cells and that inhibition of MCM2 significantly improved the therapeutic efficacy of sorafenib. Thus, MCM2 is a molecule that has potential to be used to identify sorafenib resistance and determination of MCM2 expression in a patient with HCC may help determine whether he belongs to the sorafenib-sensitive population. In trials that evaluated the efficacy of MCM2 chemical inhibitors against sorafenib, no significant toxicity was observed [42, 43]. Chemical inhibitors of MCM2, after confirming their non-toxicity towards normal cells, may also function as potential adjuvants to sorafenib.

In conclusion, this study suggests that MCM2 may play a very important role in the development of HCC. Stark contrast in the expression of MCM2 between HCC tissues and normal liver tissues strongly suggests that MCM2 may be potential diagnostic biomarker that can be used to distinguish HCC from normal tissues. The high predictive efficacy of MCM2 on prognosis also suggests that MCM2 is a biomarker of malignant biological behavior. The results of this study suggest that MCM2 regulates the stemness of its downstream molecules by enhancing hippo signaling. Since stemness plays a role in sorafenib resistance, we further discussed the effect of MCM2 on drug resistance. We found that interference of MCM2 significantly enhanced the efficacy of sorafenib, as did inhibitors of MCM2, which may provide a basis to develop solutions against sorafenib resistance.

## Supplementary information

Figure legeneds for supplementary

Original Data File

Figure S1

Figure S2

Table S1

Table S2

## Data Availability

All the data are available in the article and Supplementary Files, or available from the authors upon request.
