# Supplementary material for: MCM2 promotes the stemness and sorafenib resistance of hepatocellular carcinoma cells via hippo signaling
Source: Cell Death Discov. 2022 Oct 15;8:418. doi: 10.1038/s41420-022-01201-3 (PMC9569387; doi:10.1038/s41420-022-01201-3)
Supplement: Supplementary file 1 — Figure legeneds for supplementary [file 41420_2022_1201_MOESM1_ESM.docx]

Figure S1. Immunohistochemical assay of MCM2 in Yulin and Liuzhou cohorts and diagnostic efficacy evaluation. a-b. Expression of MCM2 in HCC, para-carcinoma and normal liver tissues in GXMU cohort in terms of immunohistochemistry assay, and the intensity of MCM2 expression was significantly higher in HCC than in para-carcinoma and liver tissue; c, The ROC analysis for MCM2 in HCC in terms of TCGA LIHC dataset, GSE14520 dataset, GXMU cohort and GSE76427 dataset.

Figure S2. Identification of down-regulation and up-regulation effects of MCM2. a, Expression of MCM2 in several HCC cell lines. b, the immunoblotting for MCM2 expression in identified cells for si-MCM2 or MCM2 overexpression, determined by western blot assay. d, the relative expression of MCM2 in identified cells for si-MCM2 or MCM2 overexpression, determined by qPCR assay. e, Corresponding histogram for primary and secondary spheroid formation in the si-MCM2 and scramble groups of the indicated cells (fig 2d). f, Corresponding histogram for colony in the si-MCM2 and scramble groups of the indicated cells (fig 2e).

Table S1. Sequence of interfering RNA for MCM2.

Table S2. Sequence of primer in this research.
