# Supplementary figures and images for: MCM2 promotes the stemness and sorafenib resistance of hepatocellular carcinoma cells via hippo signaling
Source: Cell Death Discov. 2022 Oct 15;8:418. doi: 10.1038/s41420-022-01201-3 (PMC9569387; doi:10.1038/s41420-022-01201-3)

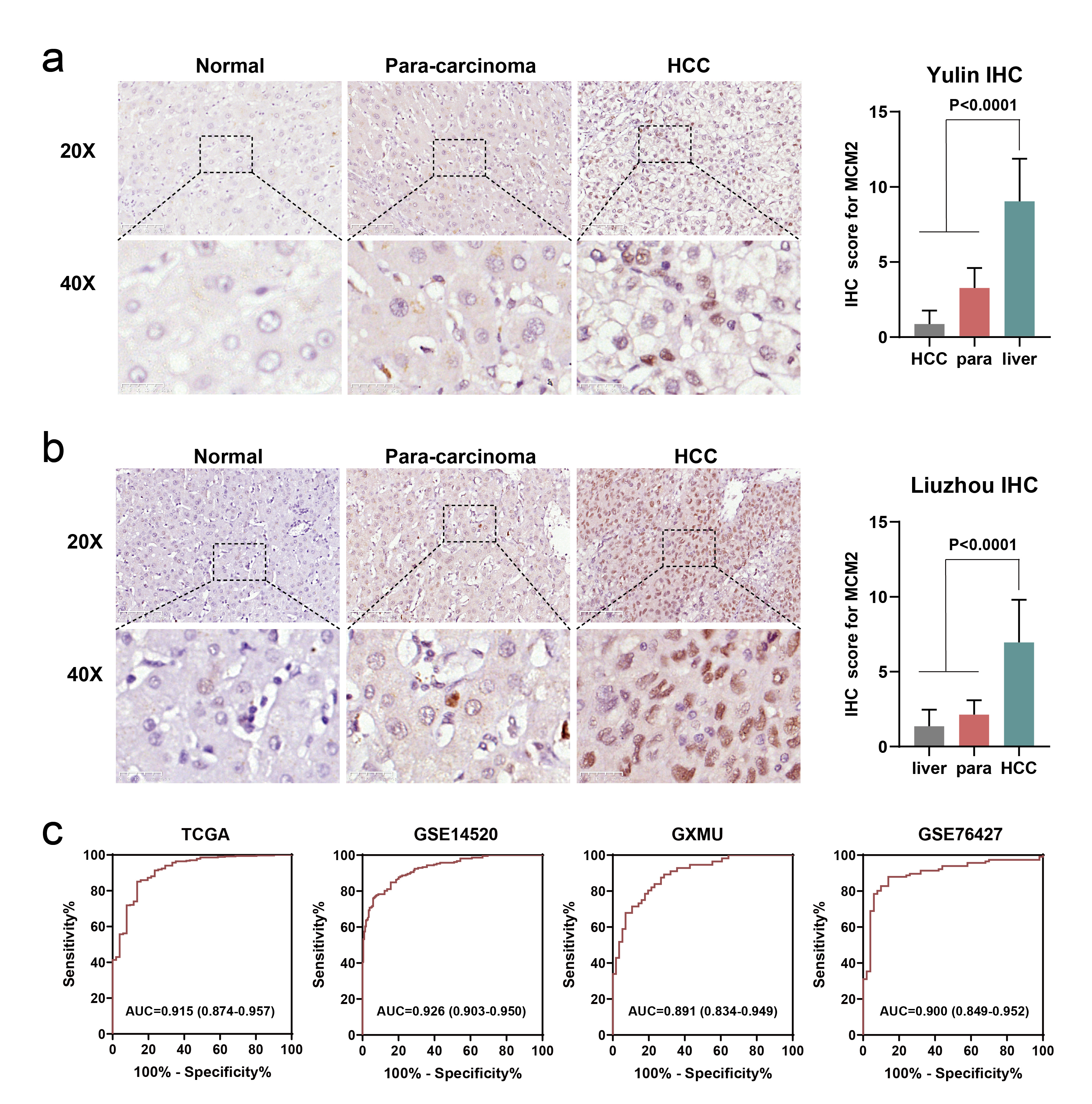

Supplement: Supplementary file 3 — Figure S1 [file 41420_2022_1201_MOESM3_ESM.jpg]

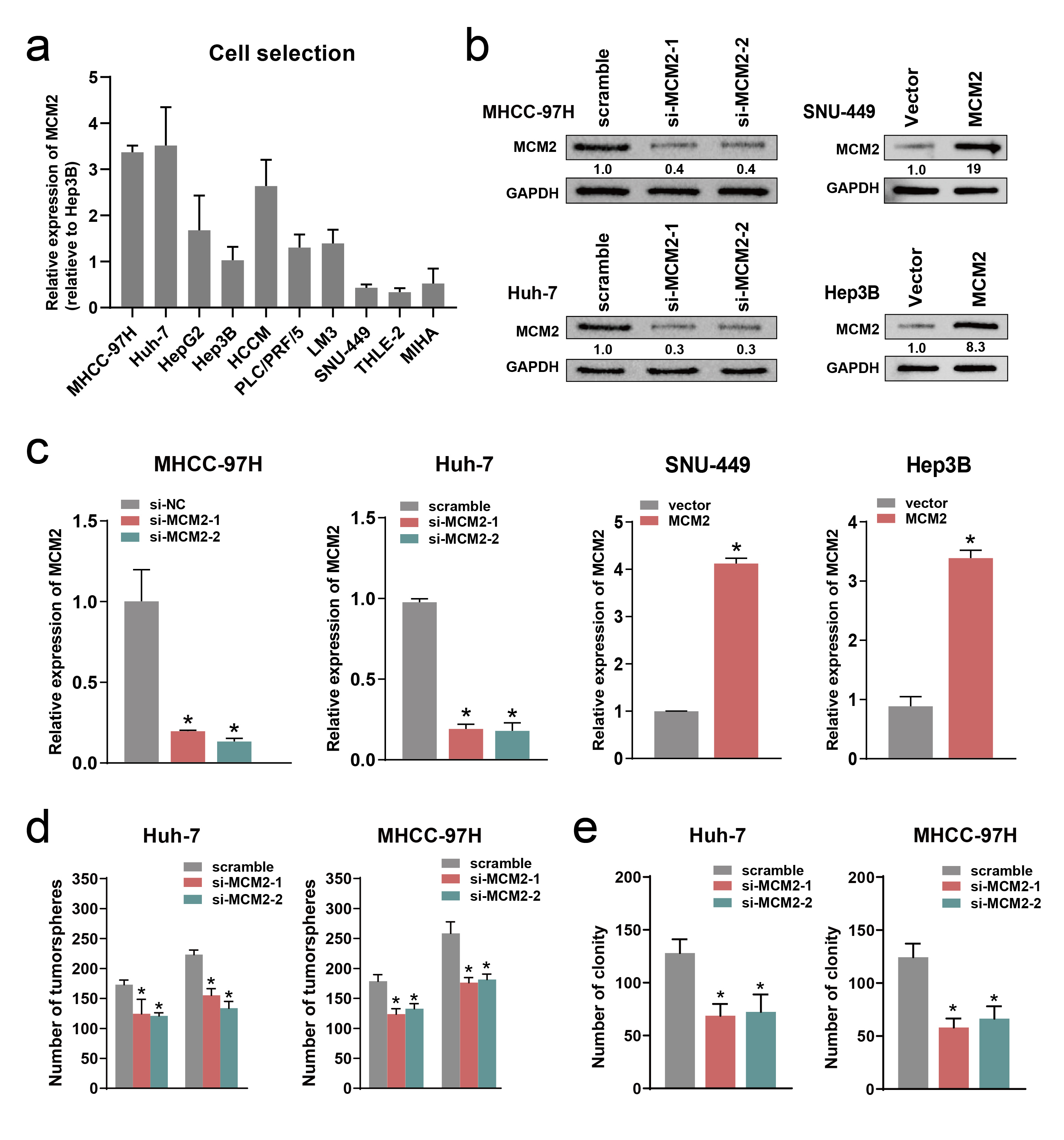

Supplement: Supplementary file 4 — Figure S2 [file 41420_2022_1201_MOESM4_ESM.jpg]
