# Supplementary material for: MCM2 promotes the stemness and sorafenib resistance of hepatocellular carcinoma cells via hippo signaling
Source: Cell Death Discov. 2022 Oct 15;8:418. doi: 10.1038/s41420-022-01201-3 (PMC9569387; doi:10.1038/s41420-022-01201-3)
Supplement: Supplementary file 5 — Table S1 [file 41420_2022_1201_MOESM5_ESM.docx]

| Oligo name |  | Sequence (5’-3’) |
| --- | --- | --- |
| hs-MCM2-si1 | sense strand | CCAAUGGCUUCCCUGUCUUTT |
|  | antisense strand | AAGACAGGGAAGCCAUUGGTT |
| hs-MCM2-si2 | sense strand | GCAUUGCUCCUUCCAUCUATT |
|  | antisense strand | UAGAUGGAAGGAGCAAUGCTT |
| hs-MCM2-si3 | sense strand | GGACAGAACCAGCAUCCAUTT |
|  | antisense strand | AUGGAUGCUGGUUCUGUCCTT |
| NC | sense strand | UUCUCCGAACGUGUCACGUTT |
|  | antisense strand | ACGUGACACGUUCGGAGAATT |
| FAM-NC | sense strand | UUCUCCGAACGUGUCACGUTT |
|  | antisense strand | ACGUGACACGUUCGGAGAATT-FAM |

Table S1. Sequence of interfering RNA for MCM2.
