# Supplementary material for: MCM2 promotes the stemness and sorafenib resistance of hepatocellular carcinoma cells via hippo signaling
Source: Cell Death Discov. 2022 Oct 15;8:418. doi: 10.1038/s41420-022-01201-3 (PMC9569387; doi:10.1038/s41420-022-01201-3)
Supplement: Supplementary file 6 — Table S2 [file 41420_2022_1201_MOESM6_ESM.docx]

| primer | sequence |
| --- | --- |
| ALDH1A1-F | CTGCCGGGAAAAGCAATCTG |
| ALDH1A1-R | GGCTATACAACACTGGCCCT |
| EPCAM-F | TGGCCGTAAACTGCTTTGTGA |
| EPCAM-R | CGTTGCACTGCTTGGCCTTA |
| GPADH-F | GGAGTCCACTGGCGTCTTCA |
| GPADH-R | GTCATGAGTCCTTCCACGATACC |
| AKT1-F | CCTCTGCTTTGTCATGGAGTACG |
| AKT1-R | AGCCCGAAGTCTGTGATCTTAAT |
| MYC-F | CTTCTCTCCGTCCTCGGATTC |
| MYC-R | AGACTCTGACCTTTTGCCAGG |
| SOX2-F | GACTTCACATGTCCCAGCACTAC |
| SOX2-R | ATTTGCTGTGGGTGATGGGATTT |
| CD133-F | CGACAATGTAACTCAGCGTCTTC |
| CD133-R | CACACAGTAAGCCCAGGTAGTAA |
| BMI1-F | TTGTCTTTTCCGCCCGCTTC |
| BMI1-R | CCTCCACAAAGCACACACATC |
| SOX9-F | GACTTCTGAACGAGAGCGAGA |
| SOX9-R | CGTTCTTCACCGACTTCCTCC |
| MMP7-F | TGCAGTGATGTATCCAACCTATG |
| MMP7-R | TTGCTAAATGGAGTGGAGGAACA |
| Slug-F | CTGGTTGCTTCAAGGACACATTAG |
| Slug-R | CAGCCAGATTCCTCATGTTTGTG |
| FGF1-F | CAGCCCTGACCGAGAAGTTTAAT |
| FGF1-R | TGGCCAGTCTCGGTACTCTTTAT |
| CCND1-F | CAGCCCTGACCGAGAAGTTTAAT |
| CCND1-R | TGGCCAGTCTCGGTACTCTTTAT |

Table S2. Sequence of primer in this research.
